# Characteristics and Health Care Use of Patients Attending Virtual Walk-in Clinics in Ontario, Canada: Cross-sectional Analysis

**DOI:** 10.2196/40267

**Published:** 2023-01-12

**Authors:** Lauren Lapointe-Shaw, Christine Salahub, Cherryl Bird, R Sacha Bhatia, Laura Desveaux, Richard H Glazier, Lindsay Hedden, Noah M Ivers, Danielle Martin, Yingbo Na, Sheryl Spithoff, Mina Tadrous, Tara Kiran

**Affiliations:** 1 Division of General Internal Medicine and Geriatrics University Health Network and Sinai Health System Toronto, ON Canada; 2 Institute of Health Policy, Management and Evaluation University of Toronto Toronto, ON Canada; 3 Department of Medicine University of Toronto Toronto, ON Canada; 4 Women's College Institute for Health System Solutions and Virtual Care Women's College Hospital Toronto, ON Canada; 5 ICES Toronto, ON Canada; 6 Support, Systems, and Outcomes Department University Health Network Toronto, ON Canada; 7 Patient partner Toronto, ON Canada; 8 Peter Munk Cardiac Centre University Health Network Toronto, ON Canada; 9 Institute for Better Health Ontario Trillium Health Partners Mississauga, ON Canada; 10 Department of Family and Community Medicine and MAP Centre for Urban Health Solutions St. Michael's Hospital Toronto, ON Canada; 11 Department of Family and Community Medicine University of Toronto Toronto, ON Canada; 12 Faculty of Health Sciences Simon Fraser University Burnaby, BC Canada; 13 Department of Family Medicine Women's College Hospital Toronto, ON Canada; 14 Women's College Research Institute Women's College Hospital Toronto, ON Canada; 15 Leslie Dan Faculty of Pharmacy University of Toronto Toronto, ON Canada

**Keywords:** virtual walk-in clinic, telemedicine, virtual care, primary health care, family practice, family physicians, Canada, health care use, emergency department, walk-in clinic, use, engagement, virtual health care, integration

## Abstract

**Background:**

Funding changes in response to the COVID-19 pandemic supported the growth of direct-to-consumer virtual walk-in clinics in several countries. Little is known about patients who attend virtual walk-in clinics or how these clinics contribute to care continuity and subsequent health care use.

**Objective:**

The objective of the present study was to describe the characteristics and measure the health care use of patients who attended virtual walk-in clinics compared to the general population and a subset that received any virtual family physician visit.

**Methods:**

This was a retrospective, cross-sectional study in Ontario, Canada. Patients who had received a family physician visit at 1 of 13 selected virtual walk-in clinics from April 1 to December 31, 2020, were compared to Ontario residents who had any virtual family physician visit. The main outcome was postvisit health care use.

**Results:**

Virtual walk-in patients (n=132,168) had fewer comorbidities and lower previous health care use than Ontarians with any virtual family physician visit. Virtual walk-in patients were also less likely to have a subsequent in-person visit with the same physician (309/132,168, 0.2% vs 704,759/6,412,304, 11%; standardized mean difference [SMD] 0.48), more likely to have a subsequent virtual visit (40,030/132,168, 30.3% vs 1,403,778/6,412,304, 21.9%; SMD 0.19), and twice as likely to have an emergency department visit within 30 days (11,003/132,168, 8.3% vs 262,509/6,412,304, 4.1%; SMD 0.18), an effect that persisted after adjustment and across urban/rural resident groups.

**Conclusions:**

Compared to Ontarians attending any family physician virtual visit, virtual walk-in patients were less likely to have a subsequent in-person physician visit and were more likely to visit the emergency department. These findings will inform policy makers aiming to ensure the integration of virtual visits with longitudinal primary care.

## Introduction

Virtual walk-in clinics provide direct-to-consumer video, phone, or text-based physician consultations, often through a mobile phone app, and typically do not have a physical location. Prior to COVID-19, virtual walk-in clinics ostensibly helped meet a primary care need for people without a family physician or those who could not access their physician in a timely way, including those in rural settings [1-3]. In Canada, Australia, and the United States, new COVID-19–related physician billing codes, intended to support virtual visits within existing primary care relationships, also drove a proliferation of virtual walk-in clinics [1,4-8]. Many patients like virtual visits, particularly with their own physician, as they do not have to take time off work, arrange childcare, travel long distances, or pay for parking [9-15].

Despite these positive perceptions, there remain concerns about the quality of care provided through virtual visits in general, and in particular the care provided by large, corporate virtual walk-in clinics [16,17]. These clinics offer an exclusively virtual experience, typically outside of existing primary care relationships, with no option for having an in-person exam [17]. The lack of a physical exam has raised questions as to whether and how virtual encounters meet the standard of care for higher-acuity presentations [18]. Exclusively virtual walk-in clinics typically do not integrate with patients’ existing sources of primary care, raising concerns about duplication and potential harm resulting from care discontinuity [17]. Virtual visits may also exacerbate inequities in access resulting from language discordance, technological access, or literacy level [16,19-21]. Additionally, the 24/7 access afforded by virtual walk-in clinics may prompt visits for transient, low-acuity medical symptoms that previously would not have occurred at all [16], raising total system costs—a phenomenon known as “supplier-induced demand” [22].

Although other studies have described the rapid expansion of virtual care, previous reports could not distinguish corporate virtual walk-in clinic visits from other virtual primary-care visits, including those with a patient’s own physician [5,23]. Little is known about the physicians and patients who use exclusively virtual walk-in clinics. Our objectives were twofold: (1) describe the family physicians working in virtual walk-in clinics and compare them to the broader family physician pool and (2) describe the characteristics and health care use of patients using virtual walk-in clinics compared to the general population and a subset that received any virtual family physician visit.

## Methods

### Study Design and Setting

We conducted a retrospective, cross-sectional study of all Ontario residents and those who had encounters at any of 13 selected virtual walk-in clinics.

Ontario is Canada’s most populous province, with over 14.5 million residents. Provincial health insurance is provided without premiums or copayments to all citizens and permanent residents and covers emergency department visits, hospitalizations, and all medically necessary physician care. Most primary care is provided by family physicians, and nearly 80% of the population is enrolled to a family physician working in a patient enrollment model [24].

Prior to April 2020, use of an approved platform (the Ontario Telemedicine Network [15]) and a video (rather than phone) visit were requirements to bill for a virtual visit. After the onset of COVID-19, the Ontario Ministry of Health introduced several new temporary physician billing codes for synchronous virtual visits by video or phone with a value equivalent to that of in-person visits (Multimedia Appendix 1 [25-32, 33 ], Tables S1A-B). Since then, the majority of publicly funded virtual visits have been conducted by phone [34,35]. Asynchronous visits (ie, provided by email or text message) are not covered by provincial insurance.

To recruit patient partners, we advertised through ICES (formerly known as the Institute for Clinical Evaluative Sciences) and selected 4 individuals with diverse backgrounds in gender, race, location, profession, and lesbian, gay, bisexual, transgender, queer/questioning, and other sexual identity (LGBTQ+) status. They also all had previous experience as patients at walk-in clinics. The patient partners, through meetings and email correspondence with the principal investigator, reviewed the analytic plan and contributed to results interpretation.

### Ethics Approval

This study was approved by the Women’s College Hospital Research Ethics Board (REB 2020-0095-E).

### Data Sources

Population-based health administrative data sets were linked using unique encoded identifiers and analyzed at ICES in Ontario, Canada (Multimedia Appendix 1, Table S2 lists the databases).

We developed a noncomprehensive list of virtual walk-in clinics by searching business names obtained from a list of group billing numbers and corresponding group names provided by the Ontario Ministry of Health. We used this list to identify all groups with “virtual” or “tele” in their name, used Google to search for the identified names, and reviewed the clinic websites to determine which provided exclusively virtual care (ie, without the possibility of an in-person office visit with a physician). In addition, we used Google to search for the combined terms “Canada” or “Ontario” AND “virtual clinic” or “telemedicine,” identifying several other groups for inclusion for a total of 20 virtual-only walk-in clinics. We then restricted the list to groups that had active billing claims during the period from April 1, 2019, to December 31, 2020 (n=13).

### Study Populations

#### Family Physicians

We included all family physicians with at least 5 virtual billing claims under one of our included virtual walk-in clinics from April 1 to December 31, 2020. The comparison group was all family physicians with active billings during this time.

#### Virtual Walk-in Clinic Patients

We selected all patients who received at least one family physician visit at 1 of the 13 included virtual walk-in clinics from April 1 to December 31, 2020. The comparison group was all Ontario residents with an active health card and a health care contact within the previous 8 years as of April 1, 2020. For measures of health care use, we restricted the Ontario population to those who had at least one virtual family physician visit from April 1 to December 31, 2020.

### Patient Characteristics and Health Care Use

We report the following patient characteristics: age, sex, neighborhood income quintile, urban or rural residence [33], and whether they were a recent provincial insurance registrant (within the past 10 years), a proxy measure for recent immigration [24]. We also examined the count of comorbidities using Johns Hopkins aggregated diagnosis groups (obtained from the Johns Hopkins ACG System, version 10) and prior health care use using adjusted clinical group (ACG) resource utilization bands (RUBs) over the previous 2 years [36]. We describe patient enrollment status, enrollment model type, and continuity of care using the Usual Provider Continuity metric [37] (Multimedia Appendix 1, Table S3 shows operational definitions of all variables).

For patients with more than one virtual walk-in clinic visit, we randomly selected one virtual walk-in clinic visit and excluded all others (Multimedia Appendix 1, Figure S1). For the Ontario population comparison, characteristics were anchored to April 1, 2020, and for characteristics that required anchoring to an encounter, we randomly selected one family physician virtual visit and excluded all others.

We report the frequencies of the top 10 most common medical diagnoses in each group. We also report whether virtual encounters were with a patient’s enrolling family physician, the encounter day of the week, and 30-day postvisit health care use, including repeat virtual visits, office visits, low-acuity emergency department visits (defined as a Canadian Triage and Acuity Scale score of 4 to 5 [38]), any emergency department visit, or urgent hospitalization.

### Data Analyses

We compared the characteristics of physicians who provided a virtual walk-in clinic visit to all family physicians with active billings. We also compared virtual walk-in clinic patients to the general Ontario population and the subset of the population that received any virtual family physician visit. Finally, we stratified health care use variables by the patients’ urban/rural residence status (large urban, small urban, or rural), because this is known to be associated with rates of emergency department use [39].

To compare groups, we used standardized mean differences (SMDs) and considered differences greater than 10% (0.1) to be significant [40]. SMDs have the advantage of quantifying the magnitude of differences between groups—this is particularly useful in studies with large sample sizes, where even very small differences can result in a *P* value <.05. To examine the adjusted association between type of virtual visit and emergency department use in the subsequent 30 days, we used logistic regression with generalized estimating equations (GEEs) to account for clustering by the index virtual visit physician. Any patient who received both types of virtual visits was removed from the “other virtual” visit group, such that each individual appeared only once. We stratified the regression by large urban, small urban, or rural residence and adjusted for patient age, sex, neighborhood income quintile, RUB, and recent provincial insurance registrant status. Observations with missing income quintile (0.2%) were not included in the regression.

Analyses were executed in SAS (version 9.4; SAS Institute Inc).

## Results

### Virtual Walk-in Clinic Volumes Over Time

**Figure 1:**
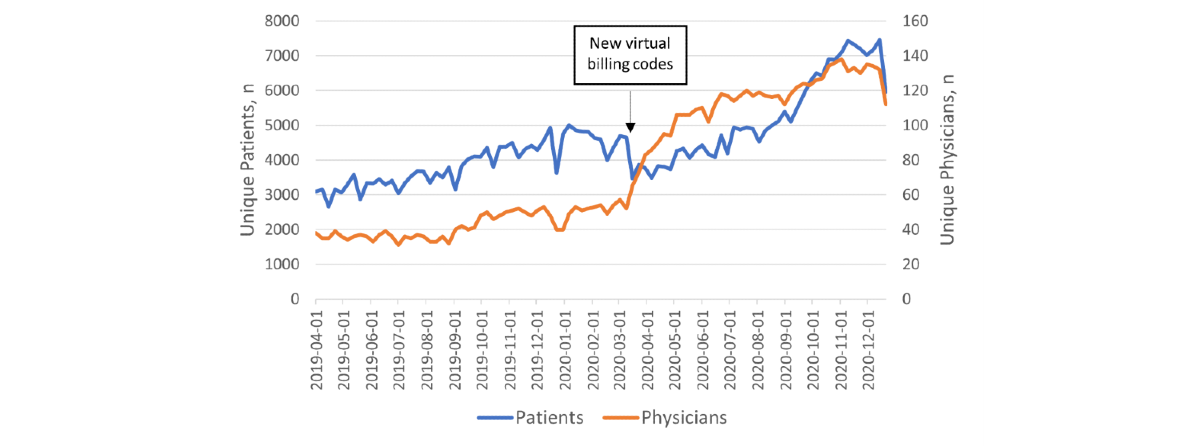
Weekly count of unique patients and unique physicians working for selected virtual walk-in clinics (n=13) in Ontario from the week beginning April 1, 2019, to the week beginning December 27, 2020. New virtual billing codes were introduced on March 14, 2020.

From April 2019 to December 2020, the weekly volume of patients increased 2-fold (Figure 1). The number of individual physicians providing virtual encounters at virtual walk-in clinics each week increased sharply between March and May 2020, and by November 2020 was 2.5 times higher than in February 2020.

### Physicians Working in Virtual Walk-in Clinics

Compared to the overall Ontario population of family physicians with active billing between April 1 and December 31, 2020 (N=14,825; Table 1), virtual walk-in clinic physicians were younger, more likely to have graduated within the past 10 years, and more likely to practice in a large urban setting. They were also considerably more likely to work fee-for-service, rather than as part of a patient enrollment model. There was no significant difference in the number of patients seen per day.

**Table 1:** Virtual walk-in clinic physician characteristics compared to all active billing family physicians. Measured between April 1, 2020, and December 31, 2020.

Physician characteristics		Provided >5 virtual walk-in clinic visits (n=242)	All family physicians with active billing (N=14,825)	Standardized mean difference^a^
**Physician age (years)**				
	Mean (SD)	40.3 (10.9)	49.3 (14.0)	0.72
	Median (IQR)	37 (32-46)	48 (38-59)	0.75
**Physician age group (years), n (%)**				
	25-34	102 (42.1)	2471 (16.7)	0.58
	35-49	95 (39.3)	5285 (35.6)	0.07
	50-64	34 (14)	4843 (32.7)	0.45
	≥65	11 (4.5)	2226 (15)	0.36
Physicians self-reporting female gender, n (%)		119 (49.2)	7112 (48)	0.02
**Time since physician graduated medical school (years), n (%)**				
	0-5	37 (15.3)	764 (5.2)	0.34
	6-10	57 (23.6)	2280 (15.4)	0.21
	11-20	32 (13.2)	2939 (19.8)	0.18
	21-30	35 (14.5)	3027 (20.4)	0.16
	≥31	20 (8.3)	4446 (30)	0.57
	Missing	61 (25.2)	1369 (9.2)	0.43
**Physician practice location, n (%)**				
	Large urban	202 (83.5)	11,010 (74.3)	0.23
	Small urban	25 (10.3)	2303 (15.5)	0.16
	Rural	9 (3.7)	1043 (7)	0.15
	Missing	6 (2.5)	469 (3.2)	0.04
**Physician primary care model, n (%)**				
	Enhanced fee-for-service	58 (24)	2753 (18.6)	0.13
	Capitation	40 (16.5)	4665 (31.5)	0.36
	Team-based	0 (0)	1824 (12.3)	0.53
	Fee-for-service (no enrollment)	136 (56.2)	4926 (33.2)	0.47
	Other	8 (3.3)	657 (4.4)	0.06
Number of patients seen per day as virtual visits, median (IQR)		12 (5-22)	13 (7-22)	0.05

^a^A standardized mean difference of at least 10% (0.1) was considered to indicate a significant difference.

### Patients Attending Virtual Walk-in Clinics

Compared to the overall Ontario population, patients who attended a virtual walk-in clinic visit were more likely to be young adults and less likely to be children or older adults (Table 2). Virtual walk-in clinic patients were also more likely to be female and live in a small urban setting. The proportion of virtual walk-in clinic patients that were new registrants or that resided in low-income neighborhoods did not differ from the overall Ontario population.

Virtual walk-in clinic patients were less likely to be enrolled to a family physician than the Ontario population (84,861/132,168, 64.2% vs 10,908,871/14,709,408, 74.2%; SMD 0.22) and had lower continuity of care (SMD 0.44). Less than 0.1% (64/132,168) of virtual walk-in visits were with the patient’s enrolling family physician.

Compared to all Ontarians who had any virtual family physician visit, virtual walk-in clinic patients had fewer comorbidities (73,526/132,168, 55.6% vs 3,207,972/6,412,304, 50% were “low”; SMD 0.11) and lower levels of previous health care use (38,584/132,168, 29.2% vs 1,358,312/6,412,304, 21.2% were “low”; SMD 0.19). They were also more likely to have their virtual visit on a Saturday or Sunday.

**Table 2:** Patient characteristics for visits at virtual walk-in clinics compared to the Ontario population with any virtual family physician visit. Measured between April 1 and December 31, 2020.

Characteristics		Visited a virtual walk-in clinic (n=132,168)	Ontario population overall (N=14,709,408)	Standardized mean difference ^a^
Age (years), mean (SD)		38.8 (19.4)	41.3 (23.0)	0.12
**Age group (years), n (%)**				
	<18	13,730 (10.4)	2,761,674 (18.8)	0.24
	18-29	35,300 (26.7)	2,217,008 (15.1)	0.29
	30-44	35,980 (27.2)	3,020,751 (20.5)	0.16
	45-64	31,714 (24)	4,065,422 (27.6)	0.08
	65-74	9744 (7.4)	1,497,270 (10.2)	0.10
	≥75	5700 (4.3)	1,147,283 (7.8)	0.15
Female sex, n (%)		79,246 (60)	7,472,638 (50.8)	0.18
**Neighborhood income quintile, n (%)**				
	Lowest (1)	29,822 (22.6)	2,890,652 (19.7)	0.07
	2	26,598 (20.1)	2,887,125 (19.6)	0.01
	3	25,667 (19.4)	2,966,912 (20.2)	0.02
	4	26,141 (19.8)	2,970,860 (20.2)	0.01
	Highest (5)	23,669 (17.9)	2,968,321 (20.2)	0.06
	Missing	271 (0.2)	25,538 (0.2)	0.01
Recent provincial insurance registrant (past 10 years), n (%)		14,334 (10.8)	1,352,790 (9.2)	0.05
**Residence, n (%)**				
	Large urban	83,484 (63.2)	10,758,196 (73.1)	0.22
	Small urban	37,581 (28.4)	2,781,005 (18.9)	0.23
	Rural	9863 (7.5)	1,025,197 (7)	0.02
	Missing	1240 (0.9)	145,010 (1)	0
**Comorbidity count, n (%)^b^**				
	Low (0-5)	73,526 (55.6)	3,207,972 (50)	0.11
	Moderate (6-9)	39,883 (30.2)	2,203,659 (34.4)	0.09
	High (≥10)	18,759 (14.2)	1,000,673 (15.6)	0.04
**Health care utilization band, n (%)^b^**				
	Low (0-2)	38,584 (29.2)	1,358,312 (21.2)	0.19
	Moderate (3)	63,896 (48.3)	3,389,803 (52.9)	0.09
	High (4-5)	29,688 (22.5)	1,664,189 (26)	0.08
Enrolled to a family physician, n (%)		84,861 (64.2)	10,908,871 (74.2)	0.22
**Primary care enrollment model type, n (%)**				
	Capitation	35,159 (26.6)	4,241,999 (28.8)	0.05
	Enhanced fee-for-service	43,114 (32.6)	4,412,144 (30.0)	0.06
	Team-based	27,508 (20.8)	3,588,850 (24.4)	0.09
	Other group	499 (0.4)	99,775 (0.7)	0.04
	Fee-for-service (no enrollment)	18,083 (13.7)	1,038,591 (7.1)	0.22
	No prior physician primary care	7805 (5.9)	1,328,049 (9)	0.12
**Continuity of care**				
	Continuity (%), median (IQR)	50 (30-83.3)	75 (50-100)	0.44
	Missing (<2 visits), n (%)	25,119 (19)	4,160,139 (28.3)	0.22
**Day of week of visit, n (%)^b^**				
	Monday	22,758 (17.2)	1,291,840 (20.1)	0.08
	Tuesday	23,089 (17.5)	1,386,411 (21.6)	0.10
	Wednesday	21,799 (16.5)	1,195,007 (18.6)	0.06
	Thursday	21,396 (16.2)	1,292,295 (20.2)	0.10
	Friday	20,539 (15.5)	887,193 (13.8)	0.05
	Saturday	12,227 (9.3)	253,311 (4)	0.21
	Sunday	10,360 (7.8)	106,247 (1.7)	0.29
Index virtual visit was with enrolling physician, n (%)^b^		64 (0)	3,949,998 (61.6)	1.79

^a^A standardized mean difference of at least 10% (0.1) was considered to indicate a significant difference.

^b^For all variables related to the index visit (including comorbidity count and health care utilization band), the Ontario population group was restricted to those with any virtual family physician visit from April 1 to December 31, 2020 (n=6,412,304).

### Top 10 Diagnoses at Virtual Encounters

Diagnoses at virtual walk-in clinic visits were similar to those for all Ontarians’ virtual family physician visits (Table 3). However, acute conditions occurred more commonly among virtual walk-in clinic visits, and chronic disease diagnoses were more common among all virtual family physician visits.

**Table 3:** Top 10 diagnoses for virtual walk-in clinic visits and for the Ontario population with any virtual family physician visit in 2020. Measured between April 1, 2020, and December 31, 2020.

Diagnoses			Values, n (%)
**Virtual walk-in visit (n=132,168)**			
	Other ill-defined conditions	13,837 (10.5)	
	Cystitis	6430 (4.8)	
	Mental health	5226 (4)	
	Acute nasopharyngitis, common cold	4838 (3.7)	
	Coronavirus	4031 (3.1)	
	Gastrointestinal symptoms^a^	3598 (2.7)	
	Other disorders of the urinary tract	3518 (2.7)	
	Essential, benign hypertension	3415 (2.6)	
	Cellulitis, abscess	2993 (2.3)	
	Family planning, contraceptive advice, advice on sterilization, abortion	2778 (2.1)	
**Ontario population with virtual family physician visit in 2020 (n=6,412,304)**			
	Mental health^b^	488,468 (7.6)	
	Other ill-defined conditions	393,541 (6.1)	
	Essential, benign hypertension	372,793 (5.8)	
	Diabetes mellitus, including complications	330,292 (5.2)	
	Musculoskeletal symptoms other than back pain^c^	225,615 (3.5)	
	Gastrointestinal symptoms^a^	202,921 (3.2)	
	Eczema, atopic dermatitis, neurodermatitis	140,262 (2.2)	
	Disorders of lipid metabolism	136,877 (2.1)	
	Acute nasopharyngitis, common cold	135,912 (2.1)	
	Lumbar strain, lumbago, coccydynia, sciatica	107,305 (1.7)	

^a^Gastrointestinal symptoms included anorexia, nausea and vomiting, heartburn, dysphagia, hiccup, hematemesis, jaundice, ascites, abdominal pain, melena, and masses.

^b^Mental health included anxiety, neurosis, hysteria, neurasthenia, obsessive compulsive neurosis, and reactive depression.

^c^Musculoskeletal symptoms other than back pain included leg cramps, leg pain, muscle pain, joint pain, arthralgia, joint swelling, and masses.

### Health Care Use Following First Virtual Visit

Patients of virtual walk-in clinics had more repeat virtual visits within 30 days than Ontarians with a virtual family physician visit (40,030/132,168, 30.3% vs 1,403,304/6,412,304, 21.9%; SMD 0.19; Table 4). They were also considerably less likely to have an in-person visit with the same physician (309/132,168, 0.2% vs 704,759/6,412,304, 11%; SMD 0.48), with any physician (15,441/132,168, 11.7% vs 980,556/6,412,304, 15.3%; SMD 0.11), or with their own physician (5,633/132,168, 4.3% vs 584,993/6,412,304, 9.1%; SMD 0.20). Patients of virtual walk-in clinics were twice as likely to have any emergency department visit (11,003/132,168, 8.3% vs 262,509/6,412,304, 4.1%; SMD 0.18), with similar results across urban/rural residence strata (Multimedia Appendix 1, Table S4). Virtual walk-in patients were also twice as likely to have a low-acuity emergency department visit (3,517/132,168, 2.7% vs 69,425/6,412,304, 1.1%; SMD 0.12).

After adjustment, those who received a virtual walk-in clinic visit remained more likely to have an emergency department visit within 30 days in all 3 urban/rural residence strata: large urban (adjusted odds ratio [aOR] 2.26, 95% CI 2.08-2.45), small urban (aOR 2.08, 95% CI 1.99-2.18), and rural locations (aOR 1.87, 95% CI 1.69-2.07).

**Table 4:** Thirty-day postvisit health care use for virtual walk-in clinic patients compared to Ontario population with a virtual family physician visit. Measured between April 1, 2020, and December 31, 2020.

Measures of use within 30 days following the virtual visit	Visit to a virtual walk-in clinic (n=132,168), n (%)	Ontario population with virtual family physician visit in 2020 (n=6,412,304), n (%)	Standardized mean difference^a^
At least one repeated virtual visit with *any* physician	40,030 (30.3)	1,403,778 (21.9)	0.19
At least one in-person visit with *same* physician	309 (0.2)	704,759 (11)	0.48
At least one in-person visit with *own enrolling* physician	5633 (4.3)	584,993 (9.1)	0.20
At least one in-person visit with *any* physician	15,441 (11.7)	980,556 (15.3)	0.11
At least one emergency department visit	11,003 (8.3)	262,509 (4.1)	0.18
At least one low-acuity emergency department visit	3517 (2.7)	69,425 (1.1)	0.12
At least one urgent hospitalization	1178 (0.9)	49,717 (0.8)	0.01

^a^A standardized difference of at least 10% (0.1) was considered to indicate a significant difference.

## Discussion

### Principal Findings

We compared patient characteristics and outcomes from visits to 13 virtual walk-in clinics to all virtual family physician visits in the Ontario population. Virtual walk-in patients were younger, were more likely to be female, and had lower continuity of care than the general population; they also had lower previous health care use than Ontario residents with any virtual family physician visit. Compared to Ontarians attending any family physician virtual visit, virtual walk-in patients were more likely to have a repeat virtual visit and less likely to have an in-person visit in the subsequent 30 days. They were also significantly more likely to visit the emergency department, a finding that held true in big cities, small towns, and rural areas, even after adjustment for potential confounders.

Our findings highlight two areas of potential concern with virtual walk-in clinics. The first is the lack of continuity of patient/physician relationships, a limitation shared with regular walk-in clinics. This is almost certainly accompanied by a lack of informational continuity, as presently there are no incentives or even regulatory frameworks compelling a virtual (or nonvirtual) walk-in physician to share information with a patient’s usual provider. Easy access to a family physician outside existing primary care relationships should be weighed against the risks of low-continuity care [41-43]. Low-continuity care does not offer opportunities for longitudinal preventive care and has been associated with more adverse events among patients with chronic conditions like diabetes [44].

The second major concern is the potential downstream consequences of a care model that operates without the possibility of a physical examination. Patients who have a virtual visit with their own family physician have more options for in-person follow-up. In the absence of a physical examination, physicians at virtual walk-in clinics may recommend that patients go to emergency departments to be examined. Alternatively, our finding of higher rates of emergency department visits among virtual walk-in clinic users could reflect the downstream consequences of an incorrect or delayed diagnosis. The absence of a physical examination also has the potential to negatively affect other dimensions of care quality [16] and lead to more inappropriate prescriptions [45-47], testing, follow-up visits [48], and referrals to consultants. Supplier-induced demand through attractive marketing campaigns, combined with increased downstream health care use, could increase overall health care costs.

Reports from the United States, United Kingdom, and Sweden have described virtual-visit users as more likely to be healthy young adults [49,50] with higher socioeconomic status [13,21,51]. Although we similarly found that users were more likely to be young adults with lower levels of health care use, our findings do not suggest that publicly funded virtual walk-in visits are disproportionately serving the affluent.

Like others [12,13], we found that virtual walk-in doctors were younger, with fewer years in practice than the average family physician. They were also more likely to be fee-for-service physicians, who would have experienced a sharp drop in income early in the pandemic due to decreased in-person visit volumes [5]. Without the income stability offered by capitation payments, fee-for-service physicians likely turned to other revenue sources, including virtual walk-in clinics.

Our 4 patient partners provided several reasons why virtual walk-in clinics may be attractive to patients. They indicated that virtual walk-in clinics are convenient and require no travel, do not require scheduling an appointment or going through a “gatekeeper” to care such as an office assistant, might be more efficient when a patient is seeking a prescription or mental health care, and also provide relative anonymity to patients seeking care if they see a different physician on each visit. For these and other reasons, patients appreciate having the choice to visit a virtual walk-in clinic.

Developing a policy landscape that favors an efficient use of virtual visits is an urgent priority for health insurers [1,52,53]. In 2021, the Ontario Ministry of Health added virtual-visit codes to the “outside use” list, which financially penalizes capitation model physicians each time their patients see other family physicians [54]. Policy makers could also consider significantly reducing the value of virtual-visit codes when used by physicians without a physical office location or without a preexisting primary care roster. Another option is for physician regulatory bodies to mandate that physicians offering virtual visits also offer in-person appointments, as was recently done in Manitoba [18].

### Limitations

Our study has several limitations. First, there are likely many more physicians and patients participating in virtual walk-in clinic care; however, because they are either not linked to a group billing number or are privately paid [4], we had no way of identifying them for study inclusion. Second, we could not distinguish video from phone visits, as these were claimed using the same billing code. We further could not capture text or email consultations, as these are ineligible for coverage by provincial insurance. Third, we exclusively focused on family physicians, as these are the most common providers of primary care in Ontario, and did not assess visits to pediatricians or psychiatrists. Finally, our findings are most generalizable to other settings with publicly funded virtual walk-in visits.

### Conclusion

The number of Ontario patients and family physicians participating in a sample of virtual walk-in clinics rose rapidly after COVID-19–related physician fee schedule changes. Our findings suggest that these visits were associated with increased emergency department use. To ensure virtual walk-in clinics contribute positively to health outcomes and health system efficiency, policy makers should prioritize regulations and billing changes that ensure the integration of virtual and in-person visits while promoting continuity of care.

## Appendix

Multimedia Appendix 1 Supplementary materials.

## Abbreviations

ACG: adjusted clinical group
aOR: adjusted odds ratio
GEE: generalized estimating equation
RUB: resource utilization band
SMD: standardized mean difference

## Data Availability

The data set from this study is held securely in coded form at ICES. While legal data sharing agreements between ICES and data providers (eg, health care organizations and government) prohibit ICES from making the data set publicly available, access may be granted to those who meet prespecified criteria for confidential access, available at www.ices.on.ca/DAS (email: das@ices.on.ca). The full data set creation plan and underlying analytic code are available from the authors upon request, understanding that the computer programs may rely upon coding templates or macros that are unique to ICES and are therefore either inaccessible or may require modification.

## References

[1] Mehrotra A, Bhatia RS, Snoswell CL (2021). Paying for telemedicine after the pandemic. JAMA.

[2] Chu C, Cram P, Pang A, Stamenova V, Tadrous M, Bhatia RS (2021). Rural telemedicine use before and during the COVID-19 pandemic: repeated cross-sectional study. J Med Internet Res.

[3] Mehrotra A, Jena AB, Busch AB, Souza J, Uscher-Pines L, Landon BE (2016). Utilization of telemedicine among rural medicare beneficiaries. JAMA.

[4] Matthewman S, Spencer S, Lavergne MR, McCracken RK, Hedden L (2021). An environmental scan of virtual "walk-in" clinics in canada: comparative study. J Med Internet Res.

[5] Glazier RH, Green ME, Wu FC, Frymire E, Kopp A, Kiran T (2021). Shifts in office and virtual primary care during the early COVID-19 pandemic in Ontario, Canada. CMAJ.

[6] Patel SY, Mehrotra A, Huskamp HA, Uscher-Pines L, Ganguli I, Barnett ML (2021). Trends in outpatient care delivery and telemedicine during the COVID-19 pandemic in the US. JAMA Intern Med.

[7] Baum A, Kaboli PJ, Schwartz MD (2021). Reduced in-person and increased telehealth outpatient visits during the COVID-19 pandemic. Ann Intern Med.

[8] Wosik J, Fudim M, Cameron B, Gellad ZF, Cho A, Phinney D, Curtis S, Roman M, Poon EG, Ferranti J, Katz JN, Tcheng J (2020). Telehealth transformation: COVID-19 and the rise of virtual care. J Am Med Inform Assoc.

[9] Dullet NW, Geraghty EM, Kaufman T, Kissee JL, King J, Dharmar M, Smith AC, Marcin JP (2017). Impact of a university-based outpatient telemedicine program on time savings, travel costs, and environmental pollutants. Value Health.

[10] Reed ME, Huang J, Graetz I, Lee C, Muelly E, Kennedy C, Kim E (2020). Patient characteristics associated with choosing a telemedicine visit vs office visit with the same primary care clinicians. JAMA Netw Open.

[11] Kelley L, Phung M, Stamenova V, Fujioka J, Agarwal P, Onabajo N, Wong I, Nguyen M, Bhatia R, Bhattacharyya O (2020). Exploring how virtual primary care visits affect patient burden of treatment. Int J Med Inform.

[12] McGrail KM, Ahuja MA, Leaver CA (2017). Virtual visits and patient-centered care: results of a patient survey and observational study. J Med Internet Res.

[13] (2019). Evaluation of Babylon GP at Hand: final evaluation report. Ipsos MORI.

[14] Rose S, Hurwitz HM, Mercer MB, Hizlan S, Gali K, Yu P, Franke C, Martinez K, Stanton M, Faiman M, Rasmussen P, Boissy A (2021). Patient experience in virtual visits hinges on technology and the patient-clinician relationship: a large survey study with open-ended questions. J Med Internet Res.

[15] Stamenova V, Agarwal P, Kelley L, Fujioka J, Nguyen M, Phung M, Wong I, Onabajo N, Bhatia RS, Bhattacharyya O (2020). Uptake and patient and provider communication modality preferences of virtual visits in primary care: a retrospective cohort study in Canada. BMJ Open.

[16] Herzer KR, Pronovost PJ (2021). Ensuring quality in the era of virtual care. JAMA.

[17] Hardcastle L, Ogbogu U (2020). Virtual care: Enhancing access or harming care?. Healthc Manage Forum.

[18] Standard of Practice: Virtual Medicine. The College of Physicians and Surgeons of Manitoba.

[19] Eberly LA, Kallan MJ, Julien HM, Haynes N, Khatana SAM, Nathan AS, Snider C, Chokshi NP, Eneanya ND, Takvorian SU, Anastos-Wallen R, Chaiyachati K, Ambrose M, O'Quinn Rupal, Seigerman M, Goldberg LR, Leri D, Choi K, Gitelman Y, Kolansky DM, Cappola TP, Ferrari VA, Hanson CW, Deleener ME, Adusumalli S (2020). Patient characteristics associated with telemedicine access for primary and specialty ambulatory care during the COVID-19 pandemic. JAMA Netw Open.

[20] Kalicki AV, Moody KA, Franzosa E, Gliatto PM, Ornstein KA (2021). Barriers to telehealth access among homebound older adults. J Am Geriatr Soc.

[21] Park J, Erikson C, Han X, Iyer P (2018). Are state telehealth policies associated with the use of telehealth services among underserved populations?. Health Aff (Millwood).

[22] Dorn SD (2021). Backslide or forward progress? Virtual care at U.S. healthcare systems beyond the COVID-19 pandemic. NPJ Digit Med.

[23] Bhatia RS, Chu C, Pang A, Tadrous M, Stamenova V, Cram P (2021). Virtual care use before and during the COVID-19 pandemic: a repeated cross-sectional study. CMAJ Open.

[24] Kiran T, Kopp A, Glazier RH (2016). Those left behind from voluntary medical home reforms in Ontario, Canada. Ann Fam Med.

[25] Glazier RH, Kopp A, Schultz SE, Kiran T, Henry DA (2012). All the right intentions but few of the desired results: lessons on access to primary care from Ontario’s patient enrolment models. Healthcare Q.

[26] (2021). ICES. Corporate Provider Database (CPDB). ICES Data Dictionary.

[27] Juurlink DN, Preyra C, Croxford R, Chong A, Austin PC, Tu JV, Laupacis A (2006). Canadian Institute for Health Information Discharge Abstract Database: A Validation Study. Institute for Clinical Evaluative Sciences.

[28] (2018). ICES. Physician Database (IPDB). ICES Data Dictionary.

[29] (2007). CIHI Data Quality Study of Ontario Emergency Department Visits for Fiscal Year 2004–2005—Executive Summary. Canadian Institute for Health Information.

[30] (2021). ICES. Ontario Health Insurance Plan (OHIP). ICES Data Dictionary.

[31] (2021). ICES. Primary Care Population (PCPOP). ICES Data Dictionary.

[32] Alter DA, Naylor CD, Austin P, Tu JV (1999). Effects of Socioeconomic Status on Access to Invasive Cardiac Procedures and on Mortality after Acute Myocardial Infarction. New England Journal of Medicine.

[33] Kralj B (2009). Measuring Rurality - RIO2008_BASIC: Methodology and Results. OMA Economics Department.

[34] Agarwal P, Wang R, Meaney C, Walji Sakina, Damji Ali, Gill Navsheer, Yip Gina, Elman Debbie, Florindo Tiffany, Fung Susanna, Witty Melissa, Pham Thuy-Nga, Ramji Noor, Kiran Tara (2022). Sociodemographic differences in patient experience with primary care during COVID-19: results from a cross-sectional survey in Ontario, Canada. BMJ Open.

[35] Kiran T, Wang R, Handford C Keeping doors open: A cross-sectional survey of family physician practice patterns during COVID-19, needs, and intentions. medRxiv.

[36] Austin P, van Walraven Carl, Wodchis W, Newman A, Anderson G (2011). Using the Johns Hopkins Aggregated Diagnosis Groups (ADGs) to predict mortality in a general adult population cohort in Ontario, Canada. Med Care.

[37] Rodriguez HP, Marshall RE, Rogers WH, Safran DG (2008). Primary care physician visit continuity: a comparison of patient-reported and administratively derived measures. J Gen Intern Med.

[38] Steele S, Anstett D, Milne WK (2008). Rural emergency department use by CTAS IV and V patients. CJEM.

[39] Kiran T, Moineddin R, Kopp A, Frymire E, Glazier RH (2018). Emergency department use and enrollment in a medical home providing after-hours care. Ann Fam Med.

[40] Austin PC (2009). Using the Standardized Difference to Compare the Prevalence of a Binary Variable Between Two Groups in Observational Research. Commun Stat Simul Comput.

[41] Pereira Gray DJ, Sidaway-Lee K, White E, Thorne A, Evans PH (2018). Continuity of care with doctors-a matter of life and death? A systematic review of continuity of care and mortality. BMJ Open.

[42] Nyweide DJ, Bynum JP (2017). Relationship between continuity of ambulatory care and risk of emergency department episodes among older adults. Ann Emerg Med.

[43] Ionescu-Ittu R, McCusker J, Ciampi A, Vadeboncoeur A, Roberge D, Larouche D, Verdon J, Pineault R (2007). Continuity of primary care and emergency department utilization among elderly people. CMAJ.

[44] Weir Daniala L, McAlister Finlay A, Majumdar Sumit R, Eurich Dean T (2016). The interplay between continuity of care, multimorbidity, and adverse events in patients with diabetes. Med Care.

[45] Ray KN, Shi Z, Gidengil CA, Poon SJ, Uscher-Pines L, Mehrotra A (2019). Antibiotic prescribing during pediatric direct-to-consumer telemedicine visits. Pediatrics.

[46] Martinez KA, Rood M, Jhangiani N, Kou L, Rose S, Boissy A, Rothberg MB (2018). Patterns of use and correlates of patient satisfaction with a large nationwide direct to consumer telemedicine service. J Gen Intern Med.

[47] Uscher-Pines L, Mulcahy A, Cowling D, Hunter G, Burns R, Mehrotra A (2016). Access and quality of care in direct-to-consumer telemedicine. Telemed J E Health.

[48] Shi Z, Mehrotra A, Gidengil CA, Poon SJ, Uscher-Pines L, Ray KN (2018). Quality of care for acute respiratory infections during direct-to-consumer telemedicine visits for adults. Health Aff (Millwood).

[49] Jain T, Mehrotra A (2020). Comparison of direct-to-consumer telemedicine visits with primary care visits. JAMA Netw Open.

[50] Alexander GC, Tajanlangit M, Heyward J, Mansour O, Qato DM, Stafford RS (2020). Use and content of primary care office-based vs telemedicine care visits during the COVID-19 pandemic in the US. JAMA Netw Open.

[51] Dahlgren C, Dackehag M, Wändell Per, Rehnberg C (2021). Determinants for use of direct-to-consumer telemedicine consultations in primary healthcare-a registry based total population study from Stockholm, Sweden. BMC Fam Pract.

[52] Bhatia RS, Jamieson T, Shaw J, Piovesan C, Kelley L, Falk W Canada's Virtual Care Revolution: A Framework for Success. CD Howe Institute.

[53] Bhatia RS, Falk W, Jamieson T, Piovesan C, Shaw J Virtual health care is having its moment. Rules will be needed. Healthy Debate.

[54] INFOBulletin 21102: Virtual Care Services and Outside Use/Access Bonus. Ontario Ministry of Health.

